# Melanosis coli: Harmless pigmentation? A case-control retrospective study of 657 cases

**DOI:** 10.1371/journal.pone.0186668

**Published:** 2017-10-31

**Authors:** Zhong Hui Liu, Dominic Chi Chung Foo, Wai Lun Law, Fion Siu Yin Chan, Joe King Man Fan, Jun Sheng Peng

**Affiliations:** 1 Department of Surgery, The Sixth Affiliated Hospital of Sun Yat-sen University, Guangzhou, Guangdong Province, China; 2 Department of Surgery, The University of Hong Kong, The University of Hong Kong—Shenzhen Hospital, Shenzhen, Guangdong Province, China; 3 Department of Surgery, Li Ka Shing Faculty of Medicine, The University of Hong Kong, Queen Mary Hospital, HKSAR, China; University Hospital Llandough, UNITED KINGDOM

## Abstract

**Backgrounds and aims:**

The association of melanosis coli with the development of colorectal polyps remains uncertain.

**Methods:**

From a total of 18263 patients who had received colonoscopy in our hospital, 219 with melanosis coli cases and 438 controls matched by age and sex (at 1:2 ratio) were included in this study. The association of incidence, number, location, and pathology of colorectal neoplasm with grades and distribution of melanosis coli were analyzed.

**Results:**

Melanosis coli was associated with significantly more colorectal polyps than control, a higher incidence of numerous colorectal polyps (number ≥ 20) (7.3% vs 0.5%; p < 0.001), and higher number of small colorectal polyps (diameter ≤ 5 mm; p < 0.01). Patients with melanosis coli had higher incidences of low-grade adenomas (31.1% vs 23.3%, p < 0.05) and non-adenoma polyps (20.1% vs 12.8%, p < 0.05) than the controls. On multivariate analysis, melanosis coli was independently associated with increased detecting rates of low grade adenoma (OR = 1.54; 95%: 1.06–2.23; p < .05), non-adenoma polyp (OR = 1.72; 95%: 1.11–2.70; p < .05) and numerous polyps (OR = 16.2, 95%: 3.66–71.6; p < .05). There was no significant difference in the incidence of high-grade adenomas or adenocarcinomas in the two population groups, but the numbers of these lesions were insufficient to permit firm conclusions. No significant differences in incidence, number, and pathology of colorectal polyps between individuals with melanosis coli of three different grades of severity were found. Melanosis located predominantly in the right colon had an interestingly lower incidence of colonic polyps in right colon than did melanosis located predominantly in the left colon or total colon (8.9% vs. 26.3%, 24.0%, p < 0.05). Patients with melanosis coli had significantly more nonspecific distal ileal ulcers than did controls (8.0% vs 0%, p < 0.001).

**Conclusion:**

Melanosis coli is associated with a higher incidence and number of colonic non-adenoma polyps and low-grade adenomas, and higher incidence of distal ileal ulcers. Melanosis coli may not be a harmless pigmentation, but a sign of chronic injury of colonic and intestinal mucosa.

## Introduction

Melanosis coli refers to the presence of brown pigment in macrophages in the mucosa of the large bowel [1, 2]. The condition, first described by Cruveilhier, in 1829 [3], and named “melanosis coli” by Virchow in 1857 [4], was initially thought to be due to the presence of melanin because the pigment reacts with the Masson-Fontana stain. However, it was soon realized that this reaction is due to cross reactivity, and the pigment is now thought to be a lipofuscin, which stains with periodic-Schiff and long Ziehl-Neelsen [1, 2]. Ultrastructural studies in animal and human tissues have demonstrated that the pigment is produced by breakdown of apoptotic colonic epithelial cells [5, 6]. The condition is mainly associated with laxative abuse, especially the anthraquinone laxatives [7–9], although it may also be seen in patients with inflammatory bowel disease [10] or chronic diarrhea [11], or with the use of non-steroidal anti-inflammatory drugs (NASIDs) [12].

The relationship of melanosis coli and neoplasia of the colonic epithelium has long been a subject of interest but remains unclarified [13, 14]. Some studies have found that patients with melanosis coli have an increased prevalence of colorectal adenomas [15], but whether this association is a causal relationship between the melanosis and the lesions or is simply due to easier detection of tiny, white colorectal polyps in the brown or black background mucosa is unknown. Thus, we have conducted this age- and sex-matched retrospective study to help explain the association between melanosis coli and colorectal polyps.

## Methods

### Study population

Eighteen thousand two hundred sixty-three patients who underwent colonoscopy in the endoscopy unit of the University of Hong Kong–Shenzhen Hospital, Shenzhen, China, from December 2012 to December 2016, were included in this study. Patients who had bad bowel preparation, incomplete colonoscopy, history of previous colorectal cancer, history of previous endoscopic polypectomy, inflammatory bowel diseases, intestinal tuberculosis, Behcet's disease, familial adenomatous polyposis, or other chronic colitis were excluded. From the remaining 15640 patients, 219 patients with melanosis coli diagnosed by colonoscopy and another 438 controls (at 1:2 ratio), based on age and sex matching, were included for analysis. Life habits, past medical history, body weight and height of patients were gathered before colonoscopy. Some new evidences have suggested dietary habit had effect on gut microbiome which would in turn affect the development of colorectal polyps. However, since there is still some argument on this, we have not collected information of patient dietary habit in this study.

### Definitions

Smoking was defined as smoking at least one cigarette per day for the previous 12 months. Alcohol consumption was defined as drinking of over 140 g of alcohol per week. Regular aspirin use was defined as aspirin use for ≥3 months during the preceding 12 months [16].

### Diagnosis and classification of melanosis coli

Colonoscopy was performed either by experienced endoscopists who had each performed more than 500 colonoscopies before the study began, or by junior endoscopists under the supervision of experienced physicians. Either CF Q260 / CF H260 (Olympus, Japan) or Fu (Fuji, Japan) colonoscopes were used. Melanosis coli was diagnosed and defined as brownish or blackish pigmentation of the colonic mucosa during colonoscopy confirmed by histology of tissue biopsy, showing pigment-laden macrophages in the lamina propria.

**Fig 1:**
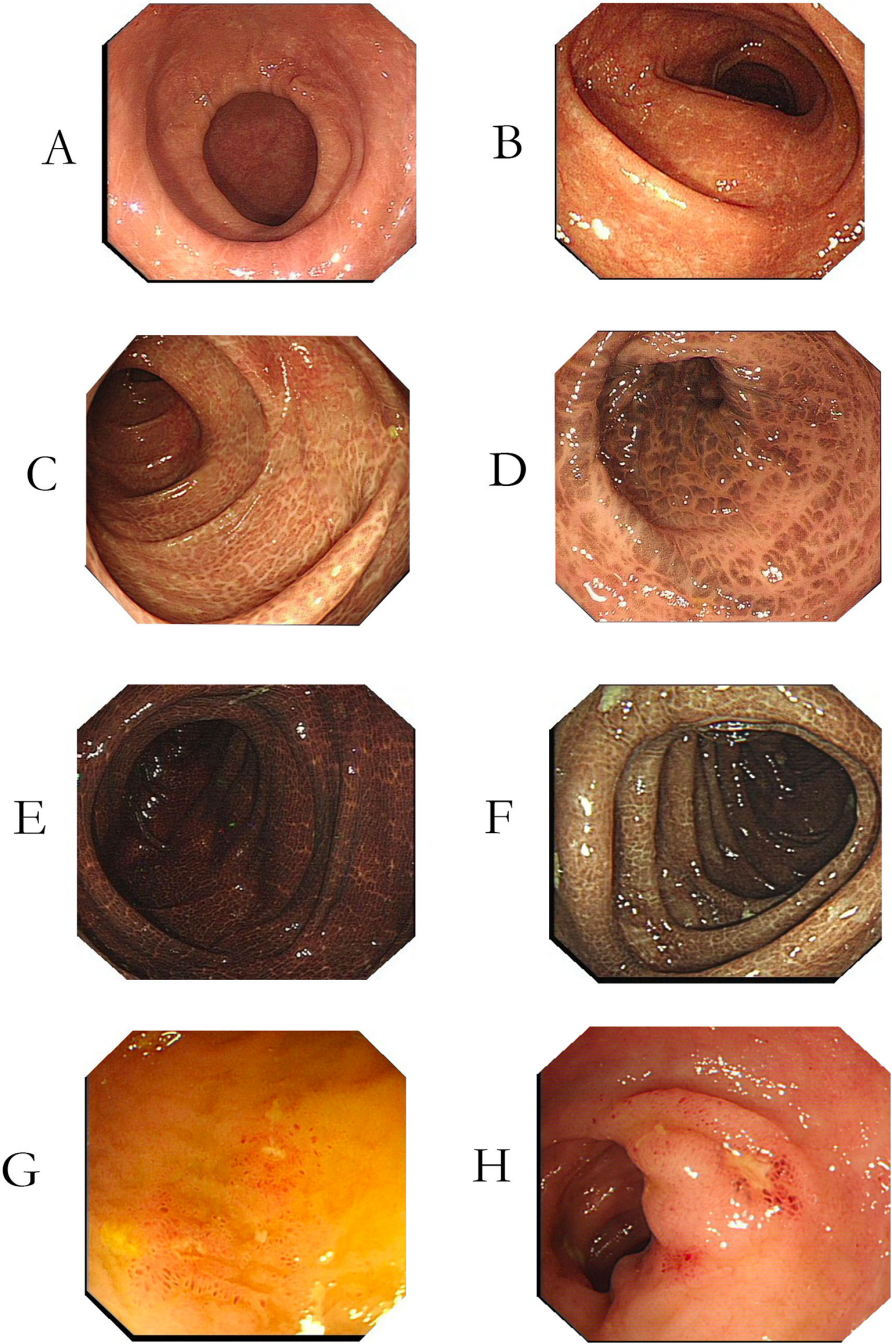
Melanosis coli of different grades and distal ileum ulcer. (A), (B): Melanosis coli Grade I, macroscopically appearing as light brown colonic mucosa, without clear boundary with normal mucosa (C), (D): Melanosis coli Grade II, macroscopically appearing as brown colonic mucosa, with clear linear or non-continuous boundary with normal mucosa. (E), (F): Melanosis coli Grade III, macroscopically appearing as dark black colonic mucosa with linear or spotted boundary with normal mucosa (G), (H): Distal ileum ulcers endoscopically discovered at six patients suffering from melanosis coli. The ulcers were multiple, small, and superficial, and mucosa around the ulcers seems normal. Pathology of the ulcers was all nonspecific inflammation.

Melanosis coli was categorized as three grades depending on the extent of pigmentation due to accumulation of pigmented phagosomes in macrophages in the lamina propria [17]: Grade I, light brown colonic mucosa with no apparent boundary with normal mucosa (Fig 1A and 1B); Grade II, brown colonic mucosa, with clear linear or non-continuous boundary with normal mucosa (Fig 1C and 1D); Grade III, dark black colonic mucosa with linear or spotted boundary with normal mucosa (Fig 1E and 1F).

The distribution of melanosis coli was defined according to the predominant region of the colon involved: Type 1, right colonic: cecum, ascending colon, and transverse colon mainly involved; Type 2, left colonic: descending colon, sigmoid colon, and rectum mainly involved; Type 3, total colonic: total colon involved.

### Diagnosis of colorectal polyps

Endoscopists performed polypectomy in our endoscopy center according to international standard: Small polyps ≤ 5mm were taken by cold biopsy forceps (CBF) or hot biopsy forceps (HBF); for polyps in diameter of 6–9mm, snare polypectomy or endoscopic mucosal resection (EMR) were performed; HSP for pedunculated polyps ≥10 mm; for sessile polyps in diameter of 10–19mm, endoscopic mucosal resection (EMR) were performed if no contraindications like non-lifting sign or suspected carcinoma in endoscopy; for sessile polyps ≥ 20mm, endoscopic submucosal dissection (ESD) or biopsy taken followed by ESD or surgical resection were performed. Tissue specimens were examined by experienced pathologists. Histopathology of the colorectal polyps was classified as three types: 1) inflammatory or hyperplastic polyp; 2) low-grade adenoma, (tubular adenoma with or without mild-to-moderate dysplasia); 3) advanced adenoma (colorectal adenoma > 10 mm in diameter, villous or tubulovillous adenoma, adenoma with high-grade dysplasia, or invasive carcinoma).

### Statistical analysis

Continuous variables are expressed as means ± SD (standard deviation). Categorical variables are expressed as number (percentage). For intergroup comparisons, continuous variables were analyzed by use of the Student’s *t* test or univariate analysis of variance, and categorical variables were analyzed by use the χ^2^ test or Fisher exact test. To identify factors independently associated with development of colorectal polyps, we performed conditional logistic regression, measuring for the association between melanosis coli and the presence of colorectal polyps, adjusting for smoking, alcohol assumption, family CRC, regular aspirin use, and indications of colonoscopy. All statistical tests were 2-sided, and a p value of less than 0.05 was considered statistically significant. Statistical analysis was carried out with licensed copy of SPSS 22.0 (SPSS, Chicago, IL, USA).

### Ethical considerations

Verbal consent was obtained from patients for research data collection and study purposes with identities kept confidential. The Institutional Review Board at the University of Hong Kong–Shenzhen Hospital approved this study (Permit Number: [2017] 108).

The laboratory protocols of this study were deposited in protocols.io: http://dx.doi.org/10.17504/protocols.io.j6rcrd6

## Results

### Associations with melanosis coli

Of the 219 patients with melanosis coli, 2 had no prior history of constipation or laxative use but had regularly taken NSAIDs for chronic headache; 8 had long-time use of both laxatives and NSAIDs; 3 had neither constipation, laxative use, nor NSAID use but had long-time consumption of Chinese traditional medicines for other diseases (chronic type B hepatitis or nephropathy). All the other 206 patients had a history of laxative abuse before colonoscopy.

### Patient demographics

Demographics of the patients and control subjects and characteristics of the melanosis coli are presented in Table 1. The 219 cases of melanosis were defined as three grades according to the classification described above: 137, Grade I (62.6%), 55, Grade II (25.1%), and 27, Grade III (12.3%). The melanosis was mainly right colonic in 56 (25.6%) patients, mainly left colonic in 38 (17.4%), and total colonic in 125 (57.1%). There were no significant differences in age, sex, family colorectal cancer history, alcohol consumption, smoking, aspirin use, and indications of colonoscopy between melanosis coli patients and control subjects. Similarly, grades and colonic distribution of melanosis were not related to any of these variables (p > .05).

**Table 1:** Demographics of patients and control subjects and characteristics of melanosis coli.

Variables	Melanosis coli (n = 219)	Control (n = 438)	*P value*	Degrees of melanosis coli			*P value*	Distribution of melanosis coli			*P value*
				Grade I (n = 137)	Grade II (n = 55)	Grade III (n = 27)		Right colon (n = 56)	Left colon (n = 38)	Total colon (n = 125)	
Age (yrs)	54.4±14.8	53.9±14.4	*0*.*69*	53.6±14.6	54.9±15.9	57.4±12.9	*0*.*60*	52.5±13.8	56.5±16.3	55.1±14.4	*0*.*34*
Female, n (%)	145 (66.2%)	290 (66.2%)	*1*.*0*	92 (67.2%)	37 (67.3%)	16 (59.3%)	*0*.*72*	38 (67.9%)	26 (68.4%)	81 (64.8%)	*0*.*88*
Current smoking, n (%)	8 (3.7%)	7 (1.6%)	*0*.*10*	2 (1.5%)	4 (7.3%)	2 (7.4%)	*0*.*11*	2 (3.6%)	2 (5.3%)	4 (3.2%)	*0*.*88*
Alcohol Consumption, n (%)	7 (3.2%)	8 (1.8%)	*0*.*27*	4 (2.9%)	2 (3.6%)	1 (3.7%)	*1*.*0*	3 (5.4%)	1 (2.6%)	3 (2.4%)	*0*.*51*
Regular aspirin use, n (%)	12 (5.5%)	19 (4.3%)	*0*.*52*	8 (5.8%)	2 (3.6%)	2 (7.4%)	*0*.*75*	3 (5.4%)	2 (5.3%)	7 (5.6%)	*1*.*0*
Family history of CRC, n (%)	8 (3.7%)	25 (5.7%)	*0*.*26*	5 (3.6%)	2 (3.6%)	1 (3.7%)	*1*.*0*	1 (1.8%)	1 (2.6%)	6 (4.8%)	*0*.*70*
Indications of colonoscopy, n (%):			*0*.*27*				*0*.*95*				*0*.*91*
Screening	84 (38.45)	158 (36.1%)		52 (38.0%)	23 (41.8%)	9 (33.3%)		21 (37.5%)	12 (31.6%)	51 (40.8%)	
Surveillance	10 (4.6%)	28 (6.4%)		8 (5.8%)	2 (3.6%)	0		4 (7.1%)	1 (2.6%)	5 (4.6%)	
Change of bowel habit	49 (22.4%)	71 (16.2%)		31 (22.6%)	12 (21.8%)	6 (22.2%)		14 (25.0%)	10 (26.3%)	25 (20.0%)	
Anemia	0	1 (0.2%)		0	0	0		0	0	0	
Per rectal bleeding	38 (17.4%)	84 (19.2%)		22 (16.1%)	10 (18.2%)	6 (22.2%)		8 (14.3%)	7 (18.4%)	23 (18.4%)	
Others	38 (17.4%)	96 (21.9%)		24 (17.5%)	8 (14.5%)	6 (22.2%)		9 (16.1%)	8 (21.1%)	21 (16.8%)	

CRC, colorectal cancer

### Association of melanosis coli with colorectal polyps

#### Association of melanosis coli with numbers of colorectal polyps

Patients with melanosis coli had significantly more colorectal polyps than did control subjects (1.05 ± 2.05 vs. 0.54 ± 0.96, *p* = .001). Moreover, as illustrated in Table 2, significantly more patients in the melanosis group (16/219, 7.3%) had numerous (number ≥ 20) colonic polyps than did control subjects (2/438, 0.5%) (odds ratio 15.9; 95% CI: 3.7–66.7, p< .001). Patients with familial adenomatous polyposis syndrome or Lynch syndrome were not included in this analysis. When cases of numerous polyps were excluded, the mean number of polyps in the melanosis patients and control subjects was significantly different only for tiny polyps, with diameters ≤ 5 mm (0.74 ± 1.66 vs. 0.35 ± 0.84, *p* = .002); the mean number of larger polyps, with diameters of 6–10 mm, >10 mm, or ≥20 mm, were similar in the two populations (p > .05), as illustrated in Table 3. We found also no significant difference in the total number of polyps or various sizes of polyps in relation to the degrees of melanosis coli or colonic distribution of the melanosis (Table 3).

**Table 2:** Melanosis coli was associated with a significantly increased incidence of numerous polyps (≥20).

Groups	*Numerous polyps (n≥ 20)*	*Odds ratio (95% CI)*	*p value*
Control (n = 438)	2 (0.5%)		< .001^*^
Melanosis coli (n = 219)	16 (7.3%)	15.9 (3.7–66.7)	

*p<0.05

**Table 3:** Association of melanosis coli with number of colorectal polyps of various sizes^a^.

Groups	Numbers of colorectal polyp in different sizes (mean ± SD)				
	*Total number*	*Diameter ≤ 5 mm*	*Diameter 6*–*9 mm*	*Diameter ≥10 mm*	*Diameter ≥20 mm*
Melanosis coli (n = 203)	1.05 ± 2.05	0.74 ± 1.66	0.27 ± 1.04	0.02 ± 0.15	0.03 ± 0.16
Control (n = 436)	0.54 ± 0.96	0.35 ± 0.84	0.13 ± 0.36	0.04 ± 0.20	0.02 ± 0.16
*p value*	0.001^*^	0.002^*^	0.06	0.38	0.60
Degrees of melanosis coli:					
Grade I (n = 128)	0.98 ±1.90	0.73 ± 1.78	0.22 ± 0.72	0.03 ± 0.17	0.02 ± 0.15
Grade II (n = 52)	1.04 ± 2.05	0.73 ± 1.39	0.23 ± 1.02	0.02 ± 0.14	0.05 ± 0.23
Grade III (n = 23)	1.46 ± 2.78	0.79 ± 1.59	0.63 ± 2.06	0	0
*p value*	0.58	0.98	0.14	0.63	0.30
Distribution of melanosis coli:					
Right colon (n = 53)	1.13 ±2.65	0.83± 2.29	0.25 ± 1.02	0.02 ± 0.13	0.02 ± 0.13
Left colon (n = 35)	1.14 ± 1.80	0.69 ± 1.53	0.14 ± 0.42	0.03 ± 0.16	0.08 ± 0.27
Total colon (n = 115)	0.98 ± 1.82	0.71 ± 1.34	0.32 ± 1.17	0.02 ± 0.15	0.02 ± 0.13
*p value*	0.87	0.89	0.98	0.25	0.10

a: Patients with numerous polyps (≥20) were excluded in this comparison.

*p<0.05

#### Association of melanosis coli with pathology of colorectal polyps

Table 4 presents the relationships between histologic type of colonic polyps and melanosis coli. Compared with control subjects, the melanosis patients had significantly higher detecting rates of inflammatory or hyperplastic polyps and low-grade adenomas (p < .05), but the detecting rate of advanced adenomas or adenocarcinoma were similar in the two populations. It is notable, however, that relatively few patients in both groups had polyps with advanced histology. There was no correlation between the three types of polyps and the degree of melanosis. A statistically significant increased number of advanced adenoma or adenocarcinoma were present in the left colon compared with the right colon or total colon (p = .04), but the numbers of these lesions were low.

**Table 4:** Association of melanosis coli with detecting rates of colonic polyps of different histological types.

Groups	Detecting rates of colonic polyps of different histological types		
	*Inflammatory or hyperplastic polyp*	*Low grade adenoma*	*Advanced adenoma or adenocarcinoma*
Melanosis coli (n = 219)	44 (20.1%)	67 (30.6%)	11 (5.0%)
Control (n = 438)	56 (12.8%)	101 (23.1%)	26 (5.9%)
*Odds ratio*, *95% CI*	**1.57 (1.10–2.25)**	**1.33 (1.03–1.73)**	0.88 (0.44–1.75)
*p value*	0.01^*^	0.04^*^	0.63
Degrees of melanosis coli:			
Grade I (n = 137)	29 (21.2%)	42 (30.7%)	7 (5.1%)
Grade II (n = 55)	9 (16.4%)	14 (25.5%)	5 (9.1%)
Grade III (n = 27)	6 (22.2%)	12 (44.4%)	0 (0%)
*p value*	0.72	0.22	0.11
Distribution of melanosis coli:			
Right colon (n = 56)	9 (16.1%)	16 (28.6%)	2 (3.6%)
Left colon (n = 38)	12 (31.6%)	10 (26.3%)	5 (13.2%)
Total colon (n = 125)	23 (18.4%)	42 (33.6%)	4 (3.2%)
*p value*	0.14	0.63	0.04^*^

*p<0.05

On multivariate conditional logistic regression, after adjusting for smoking, alcohol assumption, family CRC history, regular aspirin use and indications for colonoscopy, melanosis coli was independently associated with an increased rate of detecting numerous colonic polyps (OR = 16.2, 95%: 3.66–71.6; p < .05). (Table 5). Melanosis coli was independently associated with increased detecting rate of colonic inflammatory or hyperplastic polyp (OR = 1.72; 95%: 1.11–2.70; p < .05); and low grade colonic adenoma (OR = 1.54; 95%: 1.06–2.23; p < .05); but not independently associated with increased detecting rate of high grade colonic adenoma or adenocarcinoma (Table 5). Possibly due to limited case number of this study, other factors including smoking, alcohol assumption, family CRC history, regular aspirin use were not independently associated with detecting of colonic polyps, except family CRC history was independently associated with an increased detecting rate of low-grade adenoma (OR = 2.07; 95% CI: 1.00–4.27; p < .05). For indications of colonoscopy, only surveillance was independently associated with an increased detecting rate of low-grade adenoma (OR = 2.53; 95% CI: 1.23–5.20; p < .05). (Table 5).

**Table 5:** Multivariate analysis of risk factors for development of colonic polyps.

Multivariate Analysis				
Variables	*Inflammatory or hyperplastic polyp*	*Low grade adenoma*	*Advanced adenoma or adenocarcinoma*	*Numerous polyps (number ≥ 20)*
	*OR (95% CI)*	*OR (95% CI)*	*OR (95% CI)*	*OR (95% CI)*
Current smoking	1.12 (0.22, 5.78)	3.10 (0.88, 10.9)	2.52 (0.36, 17.90)	4.49 (0.66, 30.34)
Alcohol consumption	0.32 (0.04, 2.75)	0.42 (0.10, 1.90)	1.96 (0.28, 13.75)	0.89 (0.07, 10.8)
Family CRC history	0.31 (0.07, 1.34)	**2.06 (1.00, 4.31)** ^**b**^	1.10 (0.25, 4.85)	1.46 (0.17, 12.5)
Regular aspirin use	1.15 (0.45, 2.95)	0.71 (0.30, 1.73)	0.54 (0.07, 4.14)	1.05 (0.12, 8.91)
Indications for colonoscopy (compared with Screening)				
Surveillance	0.77 (0.28, 2.13)	**2.53 (1.23, 5.20)** ^**c**^	1.83 (0.48, 6.90)	0
Change of bowel habit	0.75 (0.40, 1.42)	0.86 (0.51, 1.46)	0.47 (0.13, 1.72)	0.68 (0.19, 2.46)
Bleeding	1.67 (0.96, 2.88)	1.45 (0.89, 2.37)	1.80 (0.75, 4.30)	0.70 (0.18, 2.72)
Others	0.66 (0.23, 1.14)	0.85 90.51, 1.43)	1.38 (0.56, 3.38)	0.46 (0.09, 2.21)
**Melanosis coli**	**1.72 (1.11, 2.70)** ^**a**^	**1.54 (1.06, 2.23)** ^**d**^	0.87 (0.42, 1.81)	**16.2 (3.66, 71.6)** ^**e**^

a, b, c, d, e: *p*<0.05

#### Association of melanosis coli with distribution of colorectal polyps

The relationship between melanosis coli and the location of colorectal polyps is shown in Table 6. Interestingly, patients with melanosis coli had a significantly higher incidence of rectal polyps than controls (p = .001). Melanosis coli also was associated with more numerous polyps in the left colon and in multiple regions (≥3) than in corresponding locations the control group (p < .05). Interestingly, subjects with melanosis coli predominantly in the right colon had a lower rate of polyps at right colon than did those with melanosis coli located predominantly in the left colon or total colon (8.9% vs. 26.3%, 24.0%; p = .04), a result that suggests that pigmentation of colonic mucosa did not have an important impact on the endoscopic detection of colorectal polyps.

**Table 6:** Association of melanosis coli with distribution of colorectal polyps.

Groups	Incidences of colorectal polyps at different sections of colon									
	*Rectum*	*Sigmoid Colon*	*Descending Colon*	*Transversal Colon*	*Ascending Colon*	*Cecum*	*Multiple sections (≥3)*	*Left Colon*		*Right Colon*
Melanosis coli (n = 219)	29 (13.2%)	49 (22.4%)	18 (8.2%)	30 (13.7%)	16 (7.3%)	7 (3.2%)	6 (2.7%)	75 (34.2%)		45 (20.5%)
Control (n = 438)	26 (5.9%)	73 (16.7%)	26 (5.9%)	40 (9.1%)	22 (5.0%)	12 (2.7%)	2 (0.5%)	114 (26.0%)		66 (15.1%)
*Odds ratio*, *95% CI*	2.23 (1.35–3.69)						5.99 (1.22–29.4)	1.32 (1.03–1.68)		
*p value*	0.001^*^	0.08	0.27	0.07	0.24	0.74	0.02^*^	0.03^*^		0.08
Degrees of melanosis coli:										
Grade I (n = 137)	17 (12.4%)	31 (22.6%)	13 (9.5%)	19 (13.9%)	9 (6.6%)	3 (2.2%)	2 (1.5%)		49 (35.8%)	27 (19.7%)
Grade II (n = 55)	9 (16.4%)	11 (20.0%)	5 (9.1%)	7 (12.7%)	5 (9.1%)	2 (3.6%)	2 (3.6%)		18 (32.7%)	12 (21.8%)
Grade III (n = 27)	3 (11.1%)	7 (25.9%)	0 (0%)	4 (14.8%)	2 (7.4%)	2 (7.4%)	2 (7.4%)		8 (29.6%)	6 (22.2%)
*p value*	0.73	0.83	0.08	0.96	0,84	0.43	0.26		0.80	0.92
Distribution of melanosis coli:										
Right colon (n = 56)	5 (8.9%)	10 (17.9%)	4 (7.1%)	3 (5.4%)	2 (3.6%)	1 (1.8%)	2 (3.6%)		15 (26.8%)	5 (8.9%)
Left colon (n = 38)	6 (15.8%)	10 (26.3%)	2 (5.3%)	6 (15.8%)	6 (15.8%)	1 (2.6%)	1 (2.6%)		14 (36.8%)	10 (26.3%)
Total colon (n = 125)	18 (14.4%)	29 (23.2%)	12 (9.6%)	21 (16.8%)	8 (6.4%)	5 (4.0%)	3 (2.4%)		46 (36.8%)	30 (24.0%)
*p value*	0.53	0.59	0.64	0.11	0.10	0.70	0.91		0.40	0.04^*^

p*<0.05

#### Melanosis coli was associated with increased incidence of distal ileum ulcer

In our center, the distal ileum is not routinely intubated during colonoscopy if no positive findings are present in the colon, although the frequency of intubation varies among endoscopists. In this series, ileal intubation was recorded in 138 patients with melanosis coli and 235 control subjects. Table 7 illustrates that ileal ulcers were found in 11 patients (8.0%) with melanosios coli but in none of the control subjects (p< .001). The ileal ulcers were multiple, small, and superficial (Fig 1G and 1H), and histologically had nonspecific inflammation. The patients with ileal ulcers had no colonic ulcers or gross inflammation and did not have evidence of chronic inflammatory bowel disease, intestinal tuberculosis, or other infectious colonic disease.

**Table 7:** Melanosis coli was associated with a significantly increased incidence of distal ileum ulcer.

Groups	*Ulcers at distal ileum*		*Odds ratio (95% CI)*	p value
	*Yes*	*No*		
Control (n = 235)	0 (0%)	235 (100%)	1.09 (1.03–1.14)	<.001*
Melanosis coli (n = 138)	11 (8.0%)	127 (92.0%)		

*p<0.05

## Discussion

In this case-control, retrospective study, conducted to help explain the association between melanosis coli and colorectal polyps, the major finding was an increased incidence of small inflammatory or hyperplastic polyps and low-grade adenomas (≤ 5 mm) in melanosis patients; the numbers of advanced adenomas or adenocarcinoma were not significantly different in patients with melanosis coli and control subjects who did not have melanosis, although the numbers of these lesions were small in both populations. We also found no significant difference in the total number of polyps and various sizes of polyps in relation to the degrees of melanosis coli or colonic distribution of the melanosis. Patients with melanosis more often had numerous polyps (≥ 20) than did control subjects.

Only a few studies have reported on the association between colorectal neoplasms and laxative use or melanosis coli. Citronberg, et al. [18] found that the risk of colorectal cancer increased with non-fiber laxative use and decreased with fiber laxative use. Nusko, et al. [19] reported that low-grade microscopic melanosis was a risk for the development of colorectal adenoma and carcinoma. However, in a retrospective cohort study [20], this group found that the adenomas in melanosis patients were smaller than those in patients without melanosis and therefore might have been more easily detected in the dark background mucosa, and were not due to an oncogenic effect of the melanosis. Unlike our study, none of these studies examined the incidence of non-adenomatous polyps in the presence of melanosis coli.

Findings in our study are some evidence against the concept that polyps are more easily seen at colonoscopy in the presence of melanosis than in its absence: Studies have demonstrated that adenomas are not pigmented, whereas non-adenomatous inflammatory and hyperplastic polyps are pigmented polyps; however, we found not only a higher incidence of neoplastic polyps but also a much higher incidence of non-neoplastic polyps in the presence of melanosis. We also found that melanosis coli located predominantly in the right colon had a lower incidence of colorectal polyps than did melanosis located predominantly in the left colon or total colon. Therefore, we feel that the high prevalence of small polyps in the setting of melanosis coli can be at least partially attributed to pathophysiologic activities of the melanosis coli itself. In support of this opinion is a report that protein levels of sonic hedgehog signaling pathway components were elevated in melanosis coli, as in colorectal cancer [21]. Many evidences have demonstrated that sonic hedgehog signaling pathway was associated with development of CRC [22–24]. The pathogenesis of melanosis coli involves apoptosis of colonic epithelial cells caused by multiple toxic factors such as anthraquinone laxative abuse. We hypothesize that toxic factors causing melanosis coli will also promote the development of colorectal polyps, including inflammatory, hyperplastic or adenomatous polyps, by chronic injury of the colonic mucosa.

An unexpected finding of this study was a high rate of distal ileal ulcers in melanosis coli patients, especially since the patients were generally healthy without evidence of inflammatory bowel disease or other intestinal inflammation, and melanic change of the small intestinal mucosa has rarely been reported. However, a case of duodenal melanosis thought to be caused by folic acid deficiency has been reported [25]. Whether the ileal ulcerations we found were causally related to the melanosis and laxative use or some other factor, such as NSAID use, is unknown. However, the possibility that anthraquinone laxative abuse is responsible is plausible since anthraquinone toxicity can damage cells, mainly through two mechanisms: 1) acting as electrophilic Michael acceptors with essential nucleophiles in the cell [26], and 2) participating in redox reactions that generate deleterious reactive oxygen species, resulting in oxidative stress [27]. We do not know whether ulcers are present in the more proximal regions of the small bowel in our patients who have distal ileal ulcers, but examination with capsule endoscopy seems justified and may be revealing.

The biological significance of the non-neoplastic or small adenomatous polyps in patients with melanosis coli is unknown. The inflammatory or hyperplastic polyps probably have little reason for concern, but the tiny adenomas may have some, albeit small, malignant potential.

Some other features of melanosis coli emerged from our study. We found that deposition of the pigmentation can occur after relatively short-term laxative use, as two of our patients had a moderate degree of melanosis seen by colonoscopy after laxative use for less than six months. Also, we found support for the opinion that melanosis coli is a reversible condition [28] since the melanosis regressed in two of our patients after laxative use was stopped and colonoscopy was repeated one year later for surveillance of colonic polyps. The reported incidence of melanosis coli seen macroscopically varies widely, from 0.25% to 5.3% [10], but the abnormal pigmentation may be seen much more often microscopically [29]. Melanosis coli has been thought to be more prominent in the proximal colon than distally, but in our series it was about equally prevalent in the left colon and was somewhat higher (57.1%) when the entire colon was involved. Elderly people are more likely to have melanosis coli; the mean age in our series was about 55 years. We suspect that the explanation for the increasing incidence of melanosis coli with aging is either more frequent constipation and laxative use or using NSAIDs more frequently to control pain and osteoarticular disorders, or increased cell apoptosis associated with aging.

We acknowledge limitations of our study. 1) The study is retrospective and thus subject to various selection biases; 2) the colonoscopies were performed by multiple endoscopists, who may have graded melanosis differently; 3) the study was conducted in a single institution; 4) indications for colonoscopic intubation of the terminal ileum may have differed among the various endoscopists; and 5) H. pylori tests were not routinely done for the patients found having ulcers in distal ileum unless concurrent upper endoscopy was performed in same session. Although some studies suspected H. pylori eradiation may be associated with clinical onset of inflammatory diseases (IBD), patients found ulcers in distal ileum in our study had no any clinical symptom or other endoscopic sign of IBD.

Despite the limitations of our study and continued uncertainty about the relationships between melanosis coli and colonic neoplasia, we feel it reasonable to recommend increased colonoscopic surveillance for patients with melanosis coli. Also, recommendation that patients discontinue the use of melanosis-inducing laxatives seems prudent.

In conclusion, patients with macroscopic melanosis coli, compared with patients who had no melanosis, had a significantly increased incidence and number of small colorectal polyps—adenomatous, inflammatory and hyperplastic. The incidence of larger (>5 mm) polyps was similar in the two populations. Patients with melanosis had a significantly higher incidence of distal ileal ulcers. Nearly all the patients with melanosis were long-time users of laxatives, especially anthroquinone laxatives. We feel that melanosis coli is not a harmless pigmentation; on the contrary, it is a sign of colonic and intestinal mucosa injury caused by toxic factors, such as anthraquinone laxative abuse. The association of melanosis coli with development of colorectal cancer remains uncertain, demanding more study in the future.

## Supporting information

S1 Table Data of melanosis coli and control. (XLSX)

## Abbreviations

CRC: Colorectal Cancer
IBD: Inflammatory Bowel Disease
NSAID: Nonsteroidal Anti-inflammatory Drug
